# Traitement chirurgical des fractures complexes de l’extrémité supérieure de l’humérus: étude rétrospective à propos de 25 cas

**DOI:** 10.11604/pamj.2020.36.5.22729

**Published:** 2020-05-06

**Authors:** Nizar Sahnoun, Sami Chtourou, Mohamed Ali Rebai, Achraf Lajmi, Mourad Hammami, Hichem Chhaydar, Yosr Hentati, Hassib Keskes

**Affiliations:** 1 Service de Chirurgie Orthopédique et Traumatologie, CHU Habib Bourguiba Sfax, Sfax, Tunisie; 2 Service de Chirurgie Orthopédique et Traumatologie, Hôpital Tataouine, Tataouine, Tunisie; 3 Département de Médecine de famille Sfax, Sfax, Tunisie; 4 Service de Radiologie CHU Hedi Chaker Sfax, Sfax, Tunisie

**Keywords:** Fracture complexe de l’extrémité supérieure de l’humérus, ostéosynthèse, prothèse, Complex fracture of the upper end of the humerus, osteosynthesis, prosthesis

## Abstract

Les fractures de l’extrémité supérieure de l’humérus posent un problème thérapeutique particulièrement pour les fractures complexes à 3 et 4 fragments. Le but de notre travail est de déterminer l’aspect épidémio-clinique des fractures complexes de l’extrémité supérieure de l’humérus chez l’adulte et d’apprécier les résultats fonctionnels et radiologiques de notre série. Il s’agit d’une série de 25 cas colligés au service d’orthopédie CHU Habib Bourguiba entre 2012 et 2017. Nous avons recensé les données épidémiologiques des patients et les circonstances du traumatisme. Le traitement était de principe chirurgical soit ostéosynthèse par plaque ou clou soit un remplacement prothétique. La réduction a été évaluée sur les radiographies post opératoires. Au recul les résultats fonctionnels ont été évalués par le score de Constant. Notre série comporte 12 hommes et 13 femmes, La moyenne d’âge de nos patients était 55 ans, les accidents de la voie publique étaient notés dans 48%, Les fractures à 4 fragments ont été retrouvées dans 76% des cas. L’ostéosynthèse par plaque vissée a été utilisée dans 40% des cas et l’enclouage antérograde a été réalisé dans 40% des cas. La prothèse a été posée pour 5 patients. Le score de constant moyen était de 65,24 avec des extrêmes allant de 35 à 88. Nous avons noté une consolidation des fractures sans cal vicieux dans 68%. Dans les fractures complexes de l’extrémité supérieure de l’humérus, une ostéosynthèse bien indiquée selon le patient et la fracture et une rééducation post opératoire précoce permettent d’avoir des résultats fonctionnels acceptables.

## Abstract (en)

Fractures of the upper end of the humerus raise a therapeutic problem in particular for three-fragment and 3 and 4-fragment complex fractures. The purpose of our study is to determine the epidemio-clinic features of complex fractures of the upper end of the humerus in adults and to assess the functional and radiological results in our case series. The study involved 25 patients whose data were collected in the Department of Orthopaedics at the Habib Bourguiba University Hospital between 2012 and 2017. We listed the epidemiological data of patients and the circumstances of the occurrence of the traumatism. Treatment was based on surgery: plate or nail osteosynthesis or prosthetics replacement. Post-operative radiographs were used to asses´ reduction. Constant-Murley score was used to assess functional outcomes at the follow-up visit. Twelve men and thirteen women were enrolled, the average age of patients was 55 years, public road accidents were reported in 48% of cases, and 4-fragment fractures were found in 76% of cases. Screwed plate osteosynthesis was used in 40% of cases and anterograde nailing was performed in 40% of cases. A prosthesis was put in place in 5 patients. Mean Constant-Murley score was 65.24, ranging between 35 years and 88 years. Consolidation of the fractures without malunions was reported in 68% of patients. In complex fractures of the upper end of the humerus, osteosynthesis performed according to patients´ needs and fracture types as well as early post-operative rehabilitation allow for acceptable functional outcomes.

Domain: Orthopedic surgery

## Introduction

Les fractures de l’extrémité supérieure de l’humérus (FESH) sont de plus en plus fréquentes. Leur incidence a triplé entre 1970 et 2002 [1]. Pour les FESH complexes le traitement est chirurgical [2] mais la prise en charge thérapeutique demeure un sujet de controverse du fait de l’absence d’un consensus bien élucidé [3]. Le but de notre travail est de déterminer l’aspect épidémio-clinique des fractures complexes de l’extrémité supérieure de l’humérus chez l’adulte et d’apprécier les résultats fonctionnels et radiologiques de notre série.

## Méthodes

**Figure 1:**
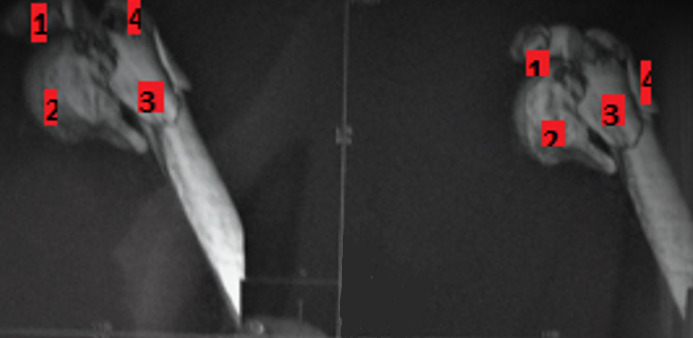
TDM de l’épaule montre une fracture à 4 fragments de l’extrémité supérieure de l’humérus

Il s’agit d’une étude rétrospective et descriptive colligée au Service de Chirurgie Orthopédique et Traumatologie au CHU Habib Bourguiba de Sfax, qui porte sur 25 cas de fractures complexes de l’extrémité supérieure de l’humérus, sur une période de 5 ans entre janvier 2012 et décembre 2017. Nous avons recensé les données cliniques des patients à savoir le sexe, l’âge au moment du traumatisme, les antécédents médico-chirurgicaux, la latéralité, le côté atteint, la profession, les circonstances du traumatisme, le mécanisme lésionnel et les lésions associées à la FESH. Le bilan radiologique a comporté 2 incidences orthogonales: une incidence d’épaule de face et un profil axillaire. Cette étude radiologique a été réalisée à 4 délais standardisés: en préopératoire, en postopératoire immédiat, au premier recul: 4 à 6 semaines après intervention et au recul final. La réalisation d’une tomodensitométrie de l’épaule a été demandée chaque fois le bilan standard est jugé insuffisant (Figure 1). Au terme de ce bilan on a défini deux groupes de fractures complexes: fracture à 3 fragments et fractures à 4 fragments. Nous avons étudié le délai de prise en charge, les différentes voies d’abord et les techniques chirurgicales adoptées: ostéosynthèse par plaque vissé, enclouage centromédullaire ou arthroplastie. Au recul le résultat fonctionnel a été évalué par le score de Constant, l’évaluation radiologique a été étudiée sur chaque incidence cherchant une consolidation en bonne position ou une pseudarthrose ou un cal vicieux en se basant sur l’évaluation de la bascule de la tête par la mesure de l’angle alpha, formé par l’intersection d’une ligne parallèle à l’axe de la diaphyse humérale et une ligne passant par le col anatomique de la tête humérale Lorsque l’angle a est compris entre 30° et 60° (45°± 15°), la tête est considérée comme non basculée de face. Au-delà de 60°, le déplacement est en valgus, et en-deçà de 30°, il est en varus. Une nécrose de la tête humérale ou arthrose a été également cherché.

## Résultats

**Figure 2:**
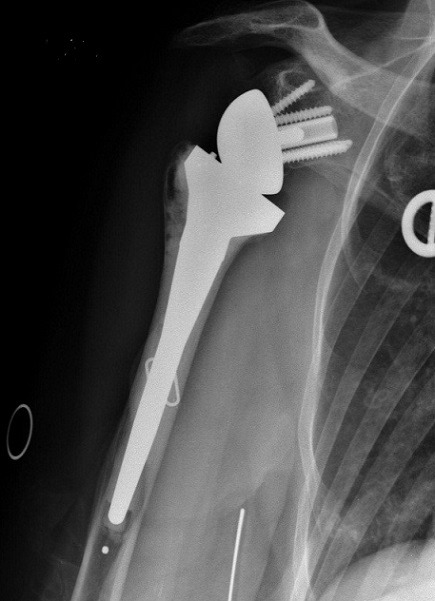
Prothèse inversée de l’épaule droite

**Figure 3:**
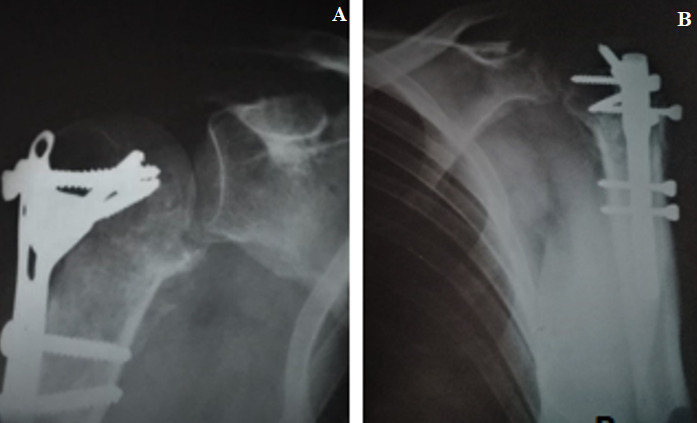
Consolidation après synthèse par plaque vissée

**Figure 4:**
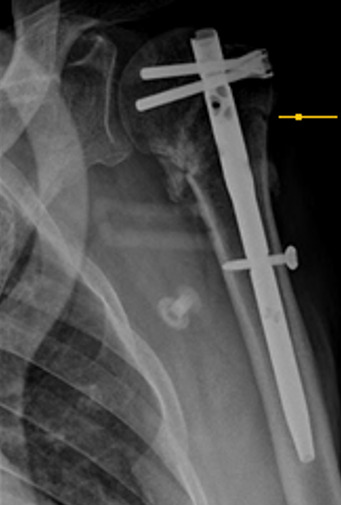
Pseudarthrose du trait de fracture métaphysaire

**Figure 5:**
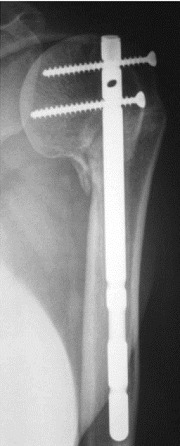
Cal vicieux: consolidation en varus minime

Notre série comporte 12 hommes et 13 femmes, la moyenne d’âge de nos patients était de 55 ans avec des extrêmes allant de 32 ans à 86 ans. On a noté deux pics de fréquence dans notre population le premier pic est compris entre 30 et 39 ans, et le deuxième, est compris entre 50 et 69 ans. Les accidents de la voie publique (AVP) étaient notés dans 48%, suivis par les accidents domestiques dans 40% des cas. Le côté non dominant a été trouvé dans 13 cas. Les lésions associées à la FESH ont intéressé le membre inférieur dans 2 cas: 1 cas de fracture de la diaphyse fémorale et 1 cas de fracture des 2 os de la jambe. Les fractures à 4 fragments ont été retrouvées chez 19 patients, soit dans 76% des cas. Nous avons trouvé 3 cas de luxations antérieuers associées aux fractures. Le délai moyen mis pour la prise en charge chirurgicale était de 14 jours. La voie d’abord delto-pectorale (Antérieure) a été utilisée chez 15 patients, soit dans 60% des cas, la voie supéro-externe (latérale) a été pratiquée chez 10 patients, soit dans 40% des cas. L’ostéosynthèse par plaque vissée a été utilisée chez 10 patients, soit dans 40% des cas et l’enclouage antérograde a été utilisé chez 10 patients, soit dans 40% des cas. La prothèse de l’épaule a été posée pour 5 patients (Figure 2). L’analyse des radiographies post opératoires a montré une réduction satisfaisante dans 80% cas, soit chez 20 patients, nous avons noté 4 cas de bascule de la tête (3 cas en valgus et un cas en varus) et un cas de translation minime de la tête. On déplore 1 cas d’infection sur le matériel d’ostéosynthèse par plaque vissée, 2 cas de migration du clou et un cas de déplacement secondaire chez des patients traités par enclouage antérograde et un cas de luxation de prothèse. La rééducation a été débutée entre J3 et j45 post opératoire. Le score de constant moyen était de 65,24 avec des extrêmes allant de 35 à 88. Pour les cas traités par plaque verrouillée, il était de 68,1 et pour les cas des prothèses, le score de constant moyen était de 60,6. Les patients traités par clou antérograde ont présenté un score de constant moyen de 66,4. Nous avons noté un taux de consolidation dans une bonne position dans 68% (Figure 3). On a noté 1 cas de pseudarthrose, 4 cas de cal vicieux: 3 cas en valgus et 1 cas en varus (Figure 4, Figure 5) et 2 cas de nécrose aseptique de la tête humérale sans avoir noter de cas d’arthrose.

## Discussion

Dans notre étude, les FESH survenaient à un âge moyen de 55 ans. Elles restent inférieures à celles rapportées dans les séries européennes et américaines [4-6] dans lesquelles, la moyenne d’âge se situe entre 60 et 72 ans. L’accroissement de la fragilité osseuse lié à l’âge est l’un des facteurs de risque prédisposant à ce type de fracture [7, 8]. dans la littérature, la prédominance féminine a été notée dans certaines séries [9, 10]. Dans notre série, les hommes étaient touchés presque autant que les femmes (12H/13F). Les patients traités par clou antérograde ont présenté un score de constant moyen de 66,4, ces résultats sont comparables à ceux rapportés par la littérature comme le montre le Tableau 1 [11-14]. Les bons résultats apparaissent être liés à l’auto-stabilité des vis de verrouillage permettant d’obtenir une stabilité de l’ostéosynthèse [13, 15, 16]. Une cicatrice dans les coiffes traversées par un clou a été visible à l’échographie dans 62% des cas de la série de Rochet dont les résultats fonctionnels étaient satisfaisants ou très satisfaisants dans 89,6% des cas [17]. Le score de constant moyen suite au traitement par plaque verrouillée était de 68,1 dans notre série. Ce score est comparable aux scores cités dans la littérature comme c’est illustré dans le Tableau 2 [6, 18-21]. En cas d’ostéoporose sévère, notre traitement de choix est le remplacement prothétique dont on a obtenu un score de constant moyen de 60,6. Dans la littérature, les résultats fonctionnels sont disparates Tableau 3 [22-25]. Ceci peut être expliqué par l’état de la coiffe des rotateurs et le type d’arthroplastie utilisé. Dans notre série, nous avons noté 04 cas de cals vicieux, il s’agissait de cal vicieux extra-articulaire à faible degré en varus, en valgus ou en translation, nous rejoignons Südkamp [19] sur le fait que le cal vicieux est secondaire à une imparfaite réduction postopératoire. La pseudarthrose a été notée dans 4% des cas, Boileau [26] et Krishnan [27] trouvent respectivement 13% et 21% des cas de pseudarthrose surtout au niveau des tubérosités.

**Tableau 1:** Score de constant pour l’enclouage

Auteurs	Score de constant
Cuny *et al* [10] 2008	62
Boudard *et al* [11] 2014	60,6
Boughebri *et al* [12] 2007	62
Doursounian *et al* [13] 2011	66
Linhart *et al* [14] 2007	82
Notre série	66,4

**Tableau 2:** Score de constant pour plaque verrouillée

Auteurs	Score de constant
Königshausen *et al* [19] 2012	66
Solberg *et al* [20] 2009	68,6
Südkamp *et al* [21] 2009	70,6
Schliemann *et al*[22] 2015	71,3
Brunner *et al* [23] 2009	72
Notre série	68,1

**Tableau 3:** Score de constant pour l’arthroplastie

Auteurs	Score de constant moyen
Gallinet et al [22] 2009	39
Bufquin et al [23] 2007	44
Potage et al [24] 2015	48,4
Boileau et al [25] 2002	54
Notre série	60,6

## Conclusion

Dans les fractures complexes de l’extrémité supérieure de l’humérus, le traitement est chirurgical dont l’objectif principal est la réduction anatomique de ces fractures complexes, une ostéosynthèse bien indiquée selon le patient et la fracture et une rééducation post opératoire précoce permettent d’avoir des résultats fonctionnels acceptables.

### État des connaissances actuelles sur le sujet

- Les fractures de l’extrémité supérieure de l’humérus sont de plus en plus fréquentes;
- Le traitement de ces fractures complexes nécessite une réduction anatomique et une ostéosynthèse stable;
- Ces fractures articulaires posent un problème de santé publique par les séquelles fonctionnelles qu’elle peut garder.

### Contribution de notre étude à la connaissance

- Une ostéosynthèse bien indiquée selon le patient et la fracture permettent d’avoir des résultats fonctionnels acceptables;
- L’évaluation de la réduction post opératoire laisse prévoir les résultats radiologiques au recul;
- La prothèse de l’épaule est le traitement de choix en cas d’ostéoporose sévère.

## Conflits d’intérêts

Les auteurs ne déclarent aucun conflit d'intérêts.
